# Increase in the prevalence of hypertension among adults exposed to the Great Chinese Famine during early life

**DOI:** 10.1186/s12199-017-0671-2

**Published:** 2017-08-25

**Authors:** Lingli Liu, Xianglong Xu, Huan Zeng, Yong Zhang, Zumin Shi, Fan Zhang, Xianqing Cao, Yao Jie Xie, Cesar Reis, Yong Zhao

**Affiliations:** 1 School of Public Health and Management, Chongqing Medical University, Chongqing, 400016 China; 2 Research Center for Medicine and Social Development, Chongqing Medical University, Chongqing, 400016 China; 3 The Innovation Center for Social Risk Governance in Health, Chongqing Medical University, Chongqing, 400016 China; 4 School of Medicine, Faculty of Health Sciences, The University of Adelaide, North Terrace, Adelaide, SA 5005 Australia; 5 School of Nursing, The Hong Kong Polytechnic University, Hong Kong, Hong Kong, Special Administrative Region of China; 6 Department of Preventive Medicine, Loma Linda University Medical Center, 24785 Stewart Street, Suite 204, Loma Linda, CA 92354 USA

**Keywords:** Hypertension, Malnutrition, Chinese famine, Adulthood, Childhood

## Abstract

**Objective:**

This study aimed to assess the association between exposure to the Great Chinese Famine (1959–1961) during early life and hypertension in adulthood.

**Methods:**

From July to September 2009, 1224 eligible adults were recruited in a cross-sectional survey using a multi-stage stratified random sampling method in Chongqing China. A questionnaire was used to collect information of hypertension and sociodemographic factors. Participants were categorized as childhood, fetal, and none exposure to famine based on the date of birth.

**Results:**

Of the sample, 12.3% reported having hypertension. The prevalence of hypertension varied by famine status: 11.9% in childhood exposure, 16.1% in fetal exposure, and 10.2% in non-exposure group. After adjusting for sociodemographic and lifestyle factors, compared with non-exposure group, fetal exposure group had an increased likelihood of having hypertension with odds ratio of 1.79 (95%CI 1.13-2.84). Although there was no significant gender and famine interaction, the positive association between famine exposure and hypertension was stronger among women than men.

**Conclusion:**

Fetal exposure to the Chinese famine may be associated with an increased risk of arthritis in adulthood in women.

## Background

Hypertension is one of the most important risk factors of cardiovascular diseases as well as other chronic diseases. Hypertension causes a significant burden to the families and the [1]. According to the 2015 *China Health and Family Planning Statistical Yearbook*, the prevalence of hypertension among adults in China increased from 25.0% in 2002 to 38% in 2012 [2]. Among adults aged above 60 years, 58.9% had hypertension in 2012 [3]. On the contrary, among adults aged 45 to 59 years the prevalence of control, treatment and awareness of hypertension was only 13.1%, 38.0% and 44.2%, respectively [3]. The direct medical cost of hypertension was estimated to be 5.7 billion dollars in 2005 in China [4]. In addition to the known risk factors of hypertension (e.g. aging, smoking, obesity, alcohol drinking, lack of physical activity and poor quality of diet), early life malnutrition may also affect hypertension.

Previous research suggests that the risk factors of chronic diseases in adulthood may originate from adverse exposures or undernutrition during fetal period [5, 6]. The Great Chinese Famine of 1959–1961 is the most extensive in human history leading to approximately over 30 million deaths [7, 8]. Emerging evidence suggests that exposure to Chinese famine in early life is related to increased risk of diabetes [9], short height [10], metabolic syndrome [5], and weight gain [11] among adults. However, the findings are criticized by a recent review as most of the findings from the published Chinese famine studies may be confounded by age [12]. Thus, there is a need to explore the association with a robust methodology.

The gender difference of hypertension in the general population is well documented but the cause is not fully understood [13]. A recent study demonstrates that women exposed to famine during fetal stage and infancy had a higher mean systolic blood pressure than those did not expose to the famine [14]. However, this finding has not been validated by other studies.

It has hypothesized that the recent rapid increase of chronic disease in China may be due to the Chinese famine in 1959–1961 [15]. As the people born during the Chinese famine are at their 50’s, a better understanding of the link between famine exposure in early life and hypertension among adults is warranted in order to manage and prevent the high burden of NCDs in China. Thus, this study aimed to assess the association of famine exposure in early life and hypertension in middle-aged population. Following the review by Li C and Lumey LH [12], we aimed to address the age related confounding by comparing fetal/infant exposure with non-exposure/childhood exposure.

## Methods

### Study design and participants

The study was conducted in Chongqing City in July 2009. A three-stage stratified random sampling method was used to recruit participants. Eligible participants were those born between 1956 and 1964 and were aged 45–53 years during time of the survey. At stage 1, 10 districts and counties were randomly selected in Chongqing City. Stage 2 involved listing eligible villages within the selected districts and counties. Approximately 8–10 villages were selected (stage 2) in each selected district/county, and 10–15 participants were randomly selected in each village (stage 3). In total, 1250 participated in the survey with a response rate of 98.4%. Of the 1250 participants, owing to missing data, six were excluded in the analysis. Detailed descriptions of the study design and methods were previously reported [16]. The study protocol was approved by the Ethics Committee of Chongqing Medical University, and its methods were carried out in accordance with the approved current guidelines. Written informed consent was obtained from all participants. All subjects were Chongqing residents who were born in 1956–1964.

### Instrument

A questionnaire survey was employed to interview people who experienced famine and were born in a specific situation of nutritional health and quality of life. The questionnaire was divided into two sections.

### Sociodemographic factors

The first section determined the general basic information of the participants, including their gender. We collected their self-reported height and weight to calculate BMI. The education level was categorized as ≤ primary school, junior middle school (basic education), ≥ senior high school (including vocational/technical secondary school and junior college), secondary education, and ≥ senior college and university (higher education). Job conditions were categorized as unemployed, employed, and stay at home, feeding procedure, and marital status was categorized as unmarried, married or cohabitation, divorce or separated, and widowed. The average monthly income was categorized as low (<¥850), medium (¥850– ¥1600) and high (>¥1601) (1 USD = ¥ 6.86 in February 2017).

### Lifestyle factors

Smoking status and alcohol drinking were categorized as yes or no. Regular physical activity and regular daily life were categorized as seldom, sometimes, or often. Sleep status was categorized as good, average, or poor. Participants were classified into low weight, normal–overweight, and obesity–BMI groups as follows: low (BMI <18.5), normal (18.5 ≤ BMI <24), overweight (24 ≤ BMI <28), and obesity (BMI ≥28) [17].

### Chinese famine exposure status

Participants were categorized into three groups based on their date of birth: (1) childhood exposure (1956–1958), (2) fetal exposure (1959–1961, 3) non-exposure: (1962–1964). Such classification was used in previous Chinese famine studies [11].

### Outcome variable

Hypertension and other health conditions were assessed by the question “Have you ever been told by a doctor or other health professional that you have (disease or condition)?” [16].

### Statistical analyses

Chi-square were used to compare differences between categorical variables. The association between famine exposure and hypertension was assessed using multivariable logistic regression. In addition to unadjusted model (model 1), two multivariable models were used. The first multivariable model adjusted for gender, education, smoking, and alcohol drinking, physical activity, sleep status, breast-feeding and diabetes. The second multivariable further adjusted for BMI. Odds ratio (OR) for hypertension was calculated for fetal exposure and childhood exposure group using non-exposure as reference group. As the non-exposure group was three years younger than the fetal group, to adjust for the potential age effect, we combined non-exposure and childhood exposure as reference group in the logistic regression to calculate the OR for hypertension among those fetal exposure group, similar to the one in International Journal of Epidemiology [12]. The statistical tests contained a two-sided test with the statistical significance set to *p* < 0.05. All data analyses were performed using statistical software (SAS version 9.1.3; SAS Institute, Cary, NC, USA).

## Results

A total of 1224 eligible respondents participated in the study. Of the participants, 150 (12.3%) reported having hypertension. Significant differences were observed on gender (*p* < 0.001), marital status (*p* = 0.0440), smoking (*p* < 0.001), alcohol drinking (*p* < 0.001), and BMI (*p* < 0.001) between individuals with and without hypertension (Table 1).

**Table 1:** Characteristics of the study participants, Chongqing, China (%)

Variable	Hypertension	Non-Hypertension	*p*-value
	(*n* = 150)	(*n* = 1074)	
Timing of exposure to famine			0.052
Childhood exposure	32.00	39.29	
Fetal and infant exposure	32.00	23.37	
Non-exposure	36.00	37.34	
Gender			<0.001**
Male	75.33	53.45	
Female	24.67	46.55	
Male			0.305
Childhood exposure	6.26	31.30	
Fetal and infant exposure	29.20	23.34	
Non-exposure	32.74	39.20	
Female			0.065
Childhood exposure	29.73	37.20	
Fetal and infant exposure	40.54	23.40	
Non-exposure	29.73	39.40	
Educational level			0.500
Basic education	16.67	15.08	
Secondary education	45.33	50.47	
Higher education	38.00	34.45	
Marital status			0.044*
Unmarried	0.67	1.21	
Married or cohabitation	88.00	92.83	
Divorce or Separated	6.67	4.38	
Widowed	4.67	1.58	
Job conditions			0.098
Unemployment	38.00	44.79	
Employed/	47.33	45.53	
Stay at home	14.67	9.68	
Average monthly income			0.116
<850 Yuan	25.33	21.88	
851 to 1600 Yuan	27.33	35.94	
>1601 Yuan	47.33	42.18	
Smoker (%)	47.33	63.31	<0.001**
Alcohol drinker (%)	28.67	45.53	<0.001**
Regular physical activity			0.469
Seldom	24.67	28.12	
Sometimes	54.00	48.70	
Usually	21.33	23.18	
Sleep status			0.108
Good	32.67	41.06	
Average	56.00	50.65	
Poor	11.33	8.29	
BMI			<0.001**
Low weight	17.33	31.38	
Normal weight	35.33	46.46	
Overweight	37.33	19.83	
Obesity	10.00	2.33	
Diabetes			0.016
No	95.33	98.51	
Yes	4.67	1.49	

Definition: **Statistical difference exists (*p* < 0.001), *with statistical difference (*P* < 0.05)

Across famine exposure groups, there were significant differences on educational level (*p* < 0.001), job conditions (*p* < 0.001), average monthly income (*p* = 0.001), and feeding practice (*p* = 0.035) (Table 2). Fetal exposure group had the lowest rate of breast-feeding in early life (75.3%) as compared with childhood (81.1%) or non-exposure group (84.7%).

**Table 2:** Characteristics of the study participants, Chongqing, China

Variable	Childhood exposure	Fetal and infant exposure	Non-exposure	*p*-value
	(*n* = 455)	(*n* = 299)	(*n* = 470)	
Age (Mean/SD)	49.69 (0.79)	47.28 (0.45)	43.44 (0.62)	<0.001**
Hypertension (%)	11.87	16.05	10.21	0.052
Men (%)	56.70	55.85	55.74	0.952
Educational level				<0.001**
Low	17.58	18.73	10.85	
Medium	50.11	54.18	46.81	
High	32.31	27.09	42.34	
Marital status				0.271
Unmarried	1.10	1.67	0.85	
Married or cohabitation	91.43	91.97	93.19	
Divorce or Separated	5.93	5.02	3.19	
Widowed	1.54	1.34	2.77	
Job conditions				<0.001**
Unemployment	47.69	36.79	44.89	
Employed/	41.76	43.48	51.06	
Stay at home	10.55	19.73	4.04	
Average monthly income				0.001*
<850 Yuan	24.40	23.08	19.79	
851 to 1600 Yuan	34.95	41.81	30.43	
>1601 Yuan	40.66	35.12	49.79	
BMI				0.346
Low weight	39.67	23.42	36.91	
Normal weight	45.59	47.16	43.40	
Overweight	20.00	22.41	23.62	
Obesity	2.86	2.01	4.47	
Smokers (%)	41.98	37.12	36.38	0.179
Alcohol drinkers (%)	58.90	52.17	57.02	0.183
Regular physical activity				0.496
Seldom	25.71	26.09	30.64	
Sometimes	50.33	50.50	47.66	
Usually	23.96	23.41	21.70	
Sleep status				0.758
Good	38.68	38.80	42.13	
Average	51.87	53.18	49.57	
Poor	9.45	8.03	8.30	
Feeding practice				0.035*
Breast feeding	81.10	75.25	84.74	
Artificial feeding	5.49	7.69	5.11	
Mixed feeding	13.41	17.06	10.43	

Definition: 1) **Statistical difference exists (*p* < 0.001), *with statistical difference (*P* < 0.05)

Fetal exposure to famine had the highest prevalence of self-reported hypertension. The prevalence of self-reported hypertension was 11.9% in childhood exposure group, 16.1% in fetal exposure group and 10.2% in non-exposure group, respectively. In multivariable logistic regression model adjusting for sociodemographic and lifestyle factors as well as BMI (model 3), compared with non-exposure group, fetal exposure group had 80% increased risk of hypertension (OR 1.79, 95% CI 1.13-2.84) (Table 3). The increased risk was seen in both genders although it was stronger in women (OR 2.34, 95% CI 1.01-5.42) than men (OR 1.67, 95% CI 0.95-2.92). Using non-exposure and childhood exposure as reference, fetal exposure group had an OR of 1.62 (95%CI 1.09-2.39) for hypertension. There was no significant increase of the likelihood of hypertension for the childhood exposure group as compared with non-exposure group.

**Table 3:** Association (odds ratio (95% CI)) between early life famine exposure and self-reported hypertension

	Childhood exposure	Fetal and infant exposure	None exposure	Fetal and infant exposure vs childhood exposure and none exposure combined
Both genders				
Model 1	1.18 (0.78-1.79)	1.68 (1.09-2.58)*	1.00	1.54 (1.07-2.24)*
Model 2	1.14 (0.75-1.74)	1.67 (1.07-2.61)*	1.00	1.60 (1.09-2.34)*
Model 3	1.24 (0.80-1.91)	1.79 (1.13-2.84)*	1.00	1.62 (1.09-2.39)*
Men				
Model 1	1.22 (0.75-1.96)	1.50 (0.89-2.51)	1.00	1.36 (0.86-2.12)
Model 2	1.17 (0.72-1.91)	1.49 ﻿(﻿0.87-2.54)	1.00	1.37 (0.86-2.17)
Model 3	1.33 (0.80-2.22)	1.67 (0.95-2.92)	1.00	1.43 (0.88-2.31)
Women				
Model 1	1.06 (0.45-2.50)	2.30 (1.02-5.17)*	1.00	2.23 (1.12-4.44)*
Model 2	1.06 (0.45-2.55)	2.37 (1.03-5.46)*	1.00	2.30 (1.12-4.69)*
Model 3	1.08 (0.45-2.59)	2.34 (1.01-5.42)	1.00	2.26 (1.10-4.64)*

*Abbreviation*: *CI* confidence intervals, *OR* odds ratio

Definition: *P < 0.05

Model 1 unadjusted

Model 2 adjusted for gender, education, smoking, alcohol drinking, physical activity, sleep status. Breast feeding and diabetes

Model 3 further adjusted for BMI

In gender combined analysis, gender was also adjusted in model 1 and model 2

## Discussion

In this population-based study, we found that fetal exposure but not childhood exposure to Chinese famine was associated with an elevated risk of self-reported hypertension in women. The findings are partly in line with previous studies. Although the relationship between early life exposure to Dutch and Leningrad Siege famine and hypertension risk is inconclusive [18–21], several studies found a positive link. Several studies in China have shown a positive association between early life famine exposure and hypertension [6, 14, 22, 23]. However, these studies have been criticized by not being adjusted the effect of age [12]. Based on the meta-analysis, Chinese famine is not associated with elevated risk of hypertension. To address the potential age confounding, we combined childhood famine exposure and non-exposure as the reference group to assess the effect of fetal famine exposure. Our finding may suggest that fetal famine exposure is associated with an increased risk of hypertension in women. The China Health and Retirement Longitudinal Study (CHARLS) indicate that nearly 40% of the adults aged 45 years and above had hypertension [24]. However, more than 40% of hypertensive patients are unaware that they are suffered from hypertension [24].

The gender difference of early life famine exposure and hypertension in our study is consistent with other studies in China. For example, the China Health and Nutrition Survey 2002 indicated that the association between famine exposure and hypertension prevalence risk was seen only among women [14]. Exposure to famine during fetal stage or early childhood exerts greater deleterious effects on female adults than male ones [14], like worsen glucose and lipid metabolism [25]. Animal studies also indicate that malnutrition may exert more adverse effects in females than males [26]. The cause of the gender difference is unknown. It could be speculated that female may suffer more than males during the famine because of son preference culture in China. More quantitative studies of larger sample are needed to further examine the relationship between fetal famine exposure and risk of hypertension among different gender.

Our study shows significant differences of educational achievement, job status, income, and early life feeding practice according to famine exposure. Early life famine exposure was associated with a lower education, income than those unexposed. This phenomenon has been shown in previous studies [27]. Negative long-term effects of impaired fetal development are not only related to health but also to educational level, employment opportunities, and income [28, 29]. Socioeconomic status is known to be associated with hypertension. It may partly explain the association between fetal exposure to famine and hypertension among adults.

The beneficial effect of breastfeeding is well known. Breastfeeding in early life can reduce the risk of hypertension in adults [30]. In the fetal exposure to famine group, the prevalence of breastfeeding is lower than other group. This may also partly explain the increase of hypertension risk among those fetal exposures to famine.

This study bears certain limitations. First, the severity of famine exposure for each individual is not known. Potentially, not all respondents’ families were equally affected by the famine. Second, we used self-reported hypertension as outcome measure. The prevalence of awareness of hypertension is low in China. This may cause serious bias. Third, the sample size was relatively small and limits the power to conduct subgroup analyses. Fourth, the cross-sectional survey data hindered the researchers to determine direct causal inferences, explore if other factors may provide better explanations on the observed relationships, and determine the direction of causal relationship. Fifth, the study sample used in the investigation was relatively homogeneous regarding race/ethnicity. Future investigations with more heterogeneous samples are warranted. In our study, the prevalence of self-reported doctor diagnosed diabetes (1.88%) was much lower than the prevalence from the 2010 Chinese national study (~11.6%) [31]. It was also lower than the prevalence of diabetes from a local study in Chongqing (4.09%) [32]. Finally, this study did not include family history of hypertension, and we were not able to adjust for factors like diabetes and dyslipidemia.

## Conclusions

Approximately 12.3% of the respondents self-reported their diagnoses of hypertension. This study suggests that fetal exposure to famine may be positively associated with hypertension in adulthood especially in women. More quantitative studies of larger sample are needed to further examine the association between fetal famine exposure and risk of hypertension among different gender.

## Abbreviations

CI: Confidence intervals
OR: Odds ratio
