# Correction to: Increase in the prevalence of hypertension among adults exposed to the great Chinese famine during early life

**DOI:** 10.1186/s12199-018-0698-z

**Published:** 2018-03-15

**Authors:** Lingli Liu, Xianglong Xu, Huan Zeng, Yong Zhang, Zumin Shi, Fan Zhang, Xianqing Cao, Yao Jie Xie, Cesar Reis, Yong Zhao

**Affiliations:** 1 School of Public Health and Management, Chongqing Medical University, Chongqing, 400016 China; 2 Research Center for Medicine and Social Development, Chongqing Medical University, Chongqing, 400016 China; 3 The Innovation Center for Social Risk Governance in Health, Chongqing Medical University, Chongqing, 400016 China; 4 School of Medicine, Faculty of Health Sciences, The University of Adelaide, North Terrace, Adelaide, SA 5005 Australia; 5 School of Nursing, The Hong Kong Polytechnic University, Hong Kong, Hong Kong, Special Administrative Region of China; 6 Department of Preventive Medicine, Loma Linda University Medical Center, 24785 Stewart Street, Suite 204, Loma Linda, CA 92354 USA

## Correction

The ‘Conclusion’ section in the Abstract was published incorrectly in the original publication of the article [1] and is corrected with this erratum as below: “Fetal exposure to the Chinese famine may be associated with an increased risk of hypertension in adulthood in women.”

Line #4 in Background on Page 1, “Hypertension causes a significant burden to the families and the society [1].”

The authors would like to correct the errors which have occurred in the ‘Table’ section, of the original publication as shown in the revised table.

Among male participants, we all use column percent in other variables, therefore, we should not use row percentage to describe “Fetal exposure” group, therefore, the authors would like to correct the error.

In ‘Table 2’ section, the number of vertical subheading “Childhood exposure (*n* = 455)” group and “Fetal and infant exposure (*n* = 299)” group was published incorrectly in the original publication of the article and are corrected with this erratum. The correct number should be “Childhood exposure (*n* = 299)” and “Fetal and infant exposure (*n* = 455)”. The underlined values in Table 1 and Table 2 have been corrected via this erratum.

**Table 1:** Characteristics of the study participants, Chongqing, China (%)

Variable	Hypertension (*n* = 150)	Non-Hypertension (*n* = 1074)	*p*-value
**Timing of exposure to famine**			0.0524
Childhood exposure	32.00	23.37	
Fetal and infant exposure	36.00	37.34	
Non-exposure	32.00	39.29	
**Gender**			< 0.001**
Male	75.33	53.45	
Female	24.67	46.55	
**Male**			0.305
Childhood exposure	29.20	23.34	
Fetal and infant exposure	38.05	37.46	
Non-exposure	32.74	39.20	
**Female**			0.065
Childhood exposure	29.73	37.20	
Fetal and infant exposure	40.54	23.40	
Non-exposure	29.73	39.40	

Definition: 1) **Statistical difference exists (*p* < 0.001), *with statistical difference (*P* < 0.05)

**Table 2:** Characteristics of the study participants, Chongqing, China

Variable	Childhood exposure (*n* = 299)	Fetal and infant exposure (*n* = 455)	Non-exposure (*n* = 470)	*p*-value
Age (Mean/SD)	49.69(0.79)	47.28(0.45)	43.44(0.62)	< 0.001**

Definition: 1) **Statistical difference exists (*p* < 0.001)
